# Diagnostic performance of simplified TI-RADS for malignant thyroid nodules: comparison with 2017 ACR-TI-RADS and 2020 C-TI-RADS

**DOI:** 10.1186/s40644-022-00478-y

**Published:** 2022-08-17

**Authors:** Zhiguang Chen, Yue Du, Linggang Cheng, Yukang Zhang, Shuai Zheng, Rui Li, Wenkai Zhang, Wei Zhang, Wen He

**Affiliations:** Department of Ultrasound, Fengtai District, Beijing Tiantan Hospital, Capital Medical University, No.119 South Fourth Ring West Road, BeijingBeijing, 100160 China

**Keywords:** Thyroid nodules, 2017 ACR-TI-RADS, 2020 C-TI-RADS, sTI-RADS

## Abstract

**Background:**

The aim of this study is to propose a new TI-RADS and compare it with the American College of Radiology (2017 ACR)-TI-RADS and the 2020 Chinese (2020 C)-TI-RADS.

**Methods:**

A retrospective analysis of 749 thyroid nodules was performed. Based on the calculated odds ratio of ultrasonic signs between benign and malignant nodules, a new thyroid nodule score and malignancy rate were calculated. A receiver operating characteristic curve was drawn to analyze the new system’s effectiveness in the differential diagnosis of benign and malignant thyroid nodules and was compared with the 2020 C-TI-RADS and 2017 ACR-TI-RADS. Five ultrasound physicians with different qualifications graded another 123 thyroid nodules according to the 2017ACR-TI-RADS, 2020 C-TI-RADS, and the newly proposed TI-RADS. Intergroup and intragroup consistency was evaluated using the Kappa test and intraclass correlation coefficient (ICC) test.

**Results:**

1) The new thyroid nodule score was divided into 0, 1, 2, 3, 4, and 5 points, with malignancy rates of 1.52%, 7.69%, 38.24%, 76.00%, 90.75%, and 93.75%, respectively. Using 3 points as the cutoff value to diagnose benign and malignant thyroid nodules, the sensitivity and specificity were 94.03% and 67.39%, respectively, which were higher than those of the 2017 ACR-TI-RADS and 2020 C-TI-RADS. The simplified TI-RADS, namely, sTI-RADS, was established as follows: sTI-RADS 3 (0 points), malignancy rate < 2%; sTI-RADS 4a (1 point), malignancy rate 2–10%; sTI-RADS 4b (2 points), malignancy rate 10–50%; sTI-RADS 4 (3 points), malignancy rate 50–90%; and sTI-RADS 5 (4 and 5 points), malignancy rate > 90%. 2) Five ultrasound doctors graded thyroid nodules by the 2017 ACR-TI-RADS, 2020C-TI-RADS and sTI-RADS. Intragroup consistency was good among all tests; ICC were 0.86 (0.82–0.90), 0.84 (0.78–0.88), and 0.88 (0.84–0.91), respectively, while only sTI-RADS had good intergroup consistency.

**Conclusion:**

In summary, we proposed a new TI-RADS, namely, sTI-RADS, which was obtained using a simple assignment method with higher specificity, accuracy, positive predictive value, and Youden index than the 2017 ACR-TI-RADS and 2020 C-TI-RADS.

## Background

Thyroid nodules are commonly seen in clinical practice, with a reported prevalence of 19–68% in the general population on high-resolution ultrasound [1, 2]. Of all nodules, only a few are malignant (approximately 7–15%) [3]. However, the incidence of thyroid cancer (TC) has increased substantially in the past 30 years, accounting for ~ 2.1% of all cancer diagnoses worldwide. Additionally, papillary thyroid microcarcinoma (PTMC) has become more common in papillary thyroid cancer (PTC) [4, 5]. Mortality has increased by 1.1% per year [6]. This evolving epidemiology may be related to biological changes in TC and changes in environmental factors. To date, the cause of TC remains unclear [7, 8]. TC is mainly diagnosed through ultrasound-guided fine-needle aspiration biopsy (FNAB) to obtain tissue or cytology for pathological examination, but the detection rate is low. When combined with gene detection, the diagnostic accuracy for *BRAF (V600E)* and *RAS* gene TC types is significantly improved [9–11].

With the development of ultrasonography and the application of FNAB, overdiagnosis and overtreatment of TC have become a concern for doctors and patients [3, 12]. Therefore, the purpose of TI-RADS is to facilitate the management of thyroid nodules [13]. Different ultrasonic signs are assigned different scores, and the thyroid nodules are classified into different categories according to the final score. According to the 2017 ACR-TI-RADS, FNAB is not recommended for TI-RADS scores 1 and 2; however, for a TI-RADS score of 4, FNAB is recommended when the nodule diameter is ≥ 1.5 cm, while follow-up is recommended for nodules ≥ 1.0 cm [14]. The 2020 C-TI-RADS is slightly different from the 2017 ACR-TI-RADS, which simplifies the process of assignment and refines the nodules classified into TI-RADS 4 as TI-RADS 4A, TI-RADS 4B, and TI-RADS 4C, with each category having its own concrete malignancy rate [15]. The assignment process of 2020 C-TI-RADS was relatively simple and had relatively higher specificity and the highest diagnostic efficiency compared with the American Thyroid Association guideline, the Korean Thyroid Association/Korean Society of Thyroid Radiology guideline, and the ACR-TI-RADS; however, when compared with the 2017 ACR-TI-RADS, the 2020 C-TI-RADS had low sensitivity [16].

To simplify the assignment process of the 2017 ACR-TI-RADS and improve the diagnostic efficiency of 2020 C-TI-RADS, we propose a new TI-RADS approach and compare it with the 2017 ACR-TI-RADS and 2020 C-TI-RADS.

## Materials and Methods

### Ethics approval and consent to participate

The Ethics Committee of Beijing Tiantan Hospital affiliated with Capital Medical University (Beijing, China) approved the study protocol. This study was conducted in accordance with the tenets of the Declaration of Helsinki.

All patients provided oral or written informed consent to participate in the study prior to biopsy.

### Study group

Data were collected from January 2018 to January 2021, and a total of 749 thyroid nodules from 627 patients were included in this study. Of them, 230 nodules were benign in 190 patients, including 43 males and 147 females with morbid ages between 20–81 years (mean age 51.76 ± 12.74 years). The mean diameter of the nodules was 2.28 ± 1.80 cm; 519 nodules were malignant in 437 patients, including 125 males and 312 females with morbid ages between 19–80 years (mean age 43.47 ± 11.99 years), and the mean diameter of the nodules was 1.04 ± 0.76 cm.

The inclusion criteria were as follows: (i) Bethesda system for reporting thyroid cytopathology was Bethesda II or Bethesda VI; (ii) the time between FNAB (or surgery) and ultrasound was no more than 2 weeks; and (iii) the image and date of ultrasound were complete and available.

The exclusion criteria were as follows: (i) Bethesda system for reporting thyroid cytopathology was Bethesda I, Bethesda III, Bethesda IV, Bethesda V; (ii) lack of ultrasonography; and (iii) incomplete image and date of ultrasound.

**Fig. 1:**
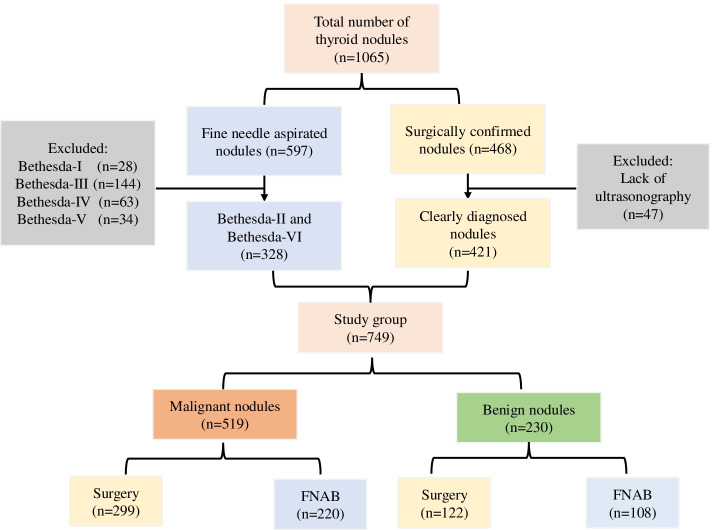
Diagram of the study group

The process of patient selection is shown in Fig. 1.

### Ultrasonography and ultrasonic signs

The patient was placed in the supine position, fully exposed to the neck, and scanned with a linear array probe. The gland where the lesion was located was carefully observed, and the size, composition, echogenicity, margin, shape, and calcification of the lesion were recorded. Two ultrasound doctors (Residents CZG and DL, with 4 and 5 years of experience, respectively) evaluated the ultrasonic signs of 749 nodules without interference with each other. In case of disagreement, senior doctors (Professor HW, with 25 years of experience) were consulted. According to the 2017 ACR-TI-RADS and 2020 C-TI-RADS [14, 15], the specific definitions of ultrasonic signs are as follows:

### Composition

1. *Solid or almost completely solid:* The nodule is entirely composed of solid tissue or solid components, accounting for more than 50% of the nodule.
2. *Mixed cystic and solid:* The nodule combines features of predominantly solid and predominantly cystic tissues.
3. *Spongiform:* The nodule is predominantly composed (> 50%) of small cystic spaces without aggregated solid tissues.
4. *Cystic or almost completely cystic:* The nodules are completely or almost completely cystic, with a thin wall or cyst accounting for > 50% of the nodules.

### Echogenicity

1. Anechoic: Applies to cystic or almost completely cystic nodules.
2. Very hypoechoic: The echogenicity was lower than that of the strap muscles.
3. Hypoechoic: The echogenicity was lower than that of the thyroid gland.
4. Isoechoic: The echogenicity is similar to that of the surrounding thyroid gland.
5. Hyperechoic: The echogenicity is higher than that of the surrounding thyroid gland.

### Shape

1. Taller-than-wide: This feature is evaluated in the axial plane by comparing the height and width of a nodule; the anteroposterior diameter of the nodule is larger than the longitudinal diameter in the longitudinal section or the transverse diameter in the transverse section.
2. Wider than tall: The anteroposterior diameter of the nodule is less than (or equal to) the longitudinal diameter in the longitudinal section or the transverse diameter in the transverse section.

### Margin

1. Smooth: The margin appears as a clear and complete curve.
2. Ill-defined: The margin is difficult to distinguish from the surrounding thyroid gland.
3. Lobulated or irregular: This refers to a spiculated or jagged edge, including spiculated, angular, or microlobulated edges.
4. Extra-thyroidal extension: This is characterized by frank invasion of the adjacent soft tissue.

### Echogenic foci

1. Microcalcifications: This refers to punctate echogenic foci of less than approximately 1 mm.
2. Large comet-tail artifacts: These refer to echogenic foci with V-shaped echoes > 1 mm with a dense tapering trail.
3. Macrocalcifications: This refers to the diameter of calcifications > 1 mm, usually accompanied by posterior shadowing.
4. Peripheral calcifications: Echogenic foci are located at the periphery of the nodules.

### Odds ratio

The odds ratio (OR) mainly refers to the ratio of exposed and nonexposed people in the case group divided by the ratio of exposed and nonexposed people in the control group.

In this study, the case group comprised malignant nodules (*n* = 519), while the control group comprised benign nodules (*n* = 230). For example, the OR of taller than wide in ultrasonic signs is equal to the ratio of people with taller than wide and wider than tall nodules in the malignant group, divided by the ratio of people with taller than wide and wider than tall nodules in the benign group.

In terms of scoring, OR = 1 indicated that this factor had no effect on the occurrence of disease; OR > 1 indicated that the factor was a risk factor; OR < 1 indicated that the factor was a protective factor.

### Prospective verification

To verify the diagnostic efficacy of the newly proposed grading system and eliminate selection bias among researchers, a total of 123 thyroid nodules confirmed as malignant or benign by biopsy or surgery from February 2021 to December 2021 were selected, and five ultrasound doctors with different qualifications (Professor ZW, with 18 years of experience; attending doctors CLG and LR, with 10 and 8 years of experience, respectively; residents ZS and ZYK, with 3 and 2 years of experience, respectively) classified the thyroid nodules according to the 2017 ACR-TI-RADS, 2020 C-TI-RADS, and the newly proposed grading system. Their diagnostic efficacy was verified using the pathological gold standard. None of them had been informed of the pathological results, and they did not interfere or consult with each other during their own evaluations.

### Statistical methods

The authors used SPSS v23 (IBM, Armonk, NY, USA). Measurement data are presented as the mean ± SD, while categorical variables are presented as frequencies.

For each ultrasonic sign, benign and malignant thyroid nodules were analyzed using single factor analysis of variance (ANOVA) and chi-square tests.

The authors reassigned these ultrasonic signs and calculated the final thyroid nodule score, which was statistically analyzed. A receiver operating characteristic (ROC) curve was drawn to calculate the best diagnostic cutoff point. The rate of unnecessary biopsies was defined as the percentage of benign nodules among the nodules currently requiring biopsy. The authors then built the sTI-RADS, calculated its sensitivity, specificity, accuracy, positive predictive value, negative predictive value, and Youden index in differentiating benign and malignant thyroid nodules, and compared it with the 2017 ACR-TI-RADS and 2020 C-TI-RADS.

The kappa test was used to analyze the diagnostic consistency of attending doctors (CLG and LR) and residents (ZS and ZYK). The ICC test was used to test the diagnostic consistency among the five ultrasound doctors, as well as the sensitivity, specificity, accuracy, positive predictive value, negative predictive value, and Youden index.

## Results

### Ultrasonic characteristics

A total of 749 thyroid nodules in 627 patients were enrolled in this study. The ORs of nodules that were solid or almost completely solid, hypoechoic, very hypoechoic, taller than wide, lobulated or irregular, or that had extrathyroidal extension, peripheral calcifications, and punctate echogenic foci were rated as greater than 1. All others were rated at less than 1.

The ORs of malignant and benign nodules were calculated and are shown in Table 1.

**Table 1:** Comparison of ultrasonic signs between the malignant nodules and benign nodules

Ultrasonic signs	Malignant nodules (***n***=519)	Benign nodules (*n*=230)	OR	*P* value
Composition				
Cystic or almost completely cystic or spongiform	1	16	0.03	0.001^f^
Mixed cystic and solid	11	70	0.05	
Solid or almost completely solid	507	144	25.23	
Echogenicity				
Anechoic	1	23	0.02	0.001^f^
Hyperechoic or isoechoic	18	90	0.06	
Hypoechoic	438	107	6.22	
Very hypoechoic	62	10	2.98	
Shape				
Wider than tall	285	202	0.17	0.001^b^
Taller than wide	234	28	5.92	
Margin				
Smooth or III-defined	53	146	0.07	NA
Lobulated or Irregular	409	84	6.46	
Extra-thyroidal extension	57	0	1.12	
Echogenic Foci				
None or large comet-tail artifacts	213	185	0.17	0.001^f^
Macrocalcifications	48	22	0.96	
Peripheral calcifications	3	1	1.33	
Punctate echogenic foci	255	22	9.13	

^b^Chi square test

^f^Continuity correction of chi square test

### The new assignment process

According to the OR value calculated from Table 1 it was found that thyroid nodules with ultrasonic signs, that were solid or almost completely solid, hypoechoic or very hypoechoic, taller than wide, lobulated, irregular or had extrathyroidal extension, and peripheral calcifications or punctate echogenic foci, were risk factors for malignant nodules (OR > 1). The authors assigned those ultrasonic signs 1 point, while the others were assigned 0 points.

**Fig. 2:**
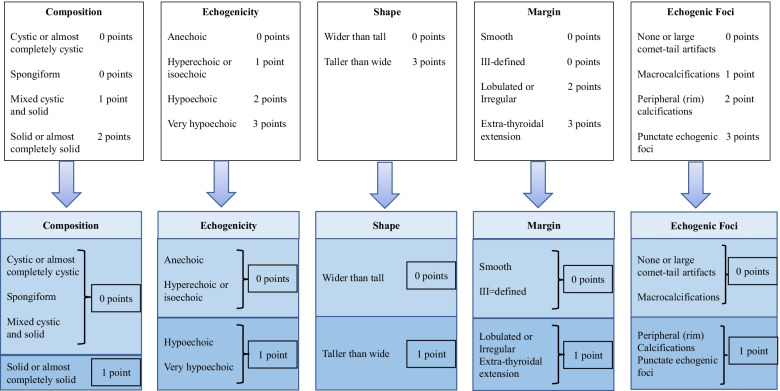
Simplified assignment process, comparison with 2017 ACR-TI-RADS

Compared with the 2017 ACR guideline, ultrasonic signs that had been assigned 2 or 3 points were now assigned 1 point, while those ultrasonic signs that had been assigned 1 point, or 0 points, were now assigned 0 points (Fig. 2).

### Comparison of the malignancy rate of thyroid nodules

According to the malignancy rate recommended by the guidelines, the authors calculated the malignancy rate of the thyroid nodules in this study and compared it with the 2017 ACR-TI-RADS and 2020 C-TI-RADS. It was suggested that the actual malignancy rate in this calculation was not exactly the same as that of the guidelines (Table 2).

**Table 2:** Comparison of malignancy risk between the various guidelines

Benign nodules		Malignant nodules	Reported malignant rate (%)	Actual malignant rate (%)
ACR-TI-RADS				
1	14	1	≤ 2	6.67
2	52	0	≤ 2	0
3	40	3	< 5	6.98
4	73	86	5—20	54.09
5	51	429	> 20	89.38
C-TI-RADS				
2	10	0	0	0
3	80	1	< 2	1.23
4A	59	26	2—10	30.59
4B	50	130	10—50	72.22
4C	31	351	50—90	91.88
5	0	11	> 90	100

### The diagnostic efficacy of the new assignment process

According to the assignment results, the new thyroid nodule scores of each patient and the malignancy rate of each score were calculated (Table 3).

**Table 3:** Nodule’s score and malignant rate of the new assignment process

(Point(s))	0	1	2	3	4	5
Benign nodules (n)	65	48	42	42	27	6
Malignant nodules (n)	1	4	26	133	265	90
Malignant rate (%)	1.52	7.69	38.24	76.00	90.75	93.75

**Fig. 3:**
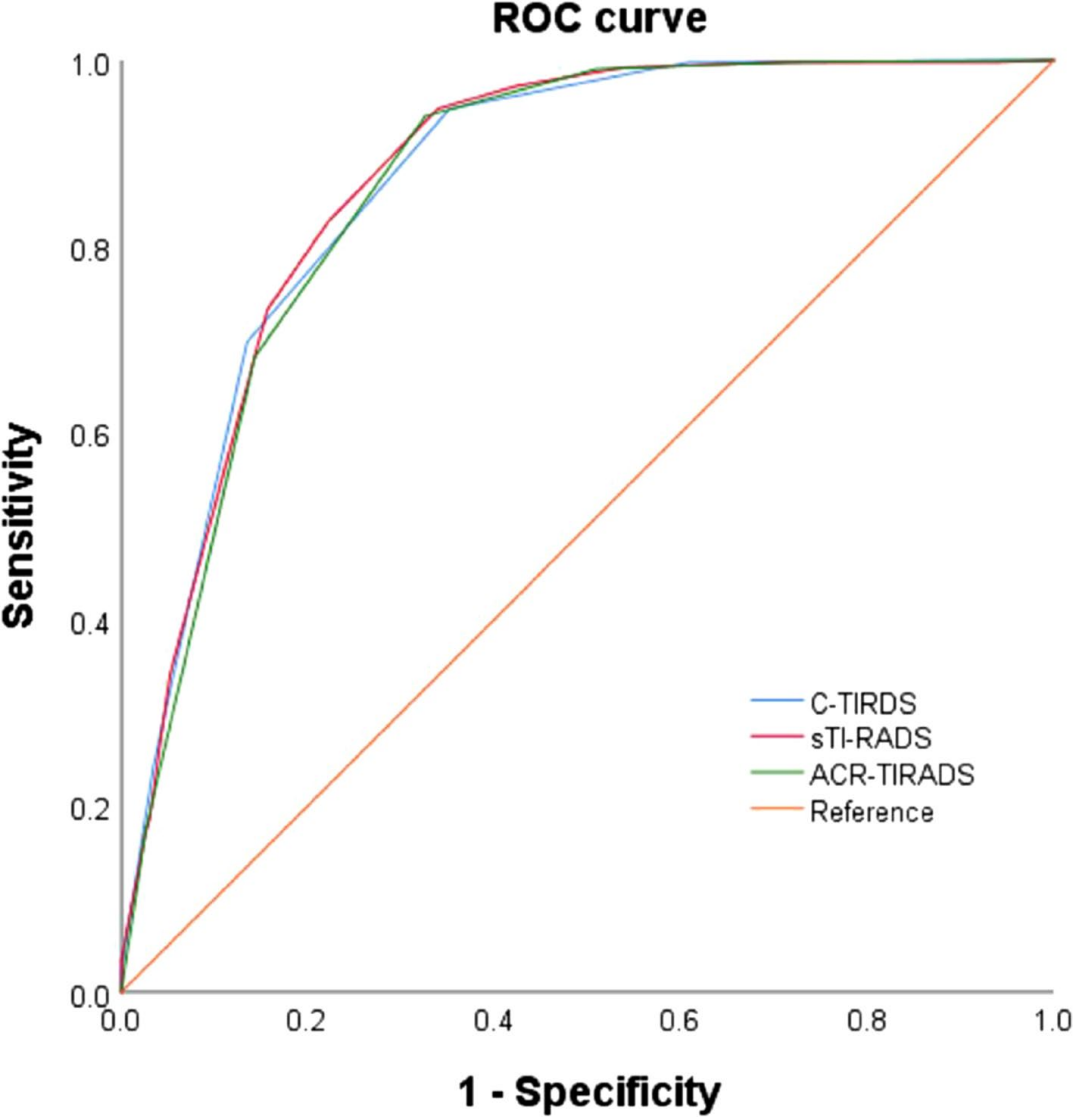
ROC of the new assignment process, 2020 C-TIRADS, and 2017 ACR-TIRADS to diagnose benign and malignant thyroid nodules

Then, an ROC curve was created (Fig. 3). The area under the ROC curve (AUC) was 0.867 (95% confidence interval, 0.836–0.898). Using 3 points as the best cutoff to diagnose benign and malignant thyroid nodules, the sensitivity and specificity were 94.03% and 67.39%, respectively.

### The simplified TI-RADS (sTI-RADS) was proposed and compared with the 2020 C-TI-RADS

According to the scoring results of the thyroid nodules, the authors referred to the 2020 C-TI-RADS grading system and classified 0 points as sTI-RADS 3, with a malignancy rate of < 2%. One point was classified as sTI-RADS 4a, with a malignancy rate of 2–10%; 2 points as sTI-RADS 4b, with a malignancy rate of 10–50%; 3 points as sTI-RADS 4c, with a malignancy rate of 50–90%; and 4 and 5 points as sTI-RADS 5, with a malignancy rate of > 90%.

**Fig. 4:**
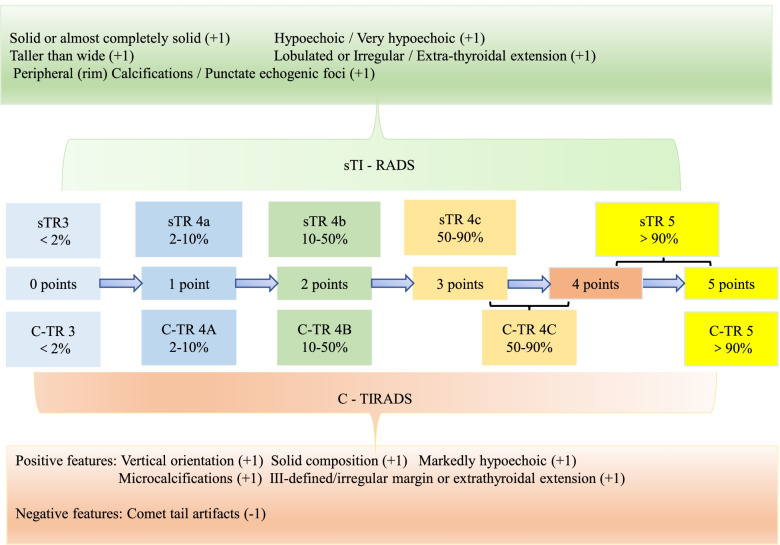
The assignment process of sTI-RADS, and comparison with 2020 C-TI-RADS

In contrast to the 2020 C-TI-RADS, 4 and 5 points were classified into five categories of sTI-RADS, and the malignancy rate was > 90%, while 3 and 4 points were classified into 4C categories, and 5 points were classified into 5 categories of 2020 C-TI-RADS (Fig. 4).

An “almost completely solid” composition designation was added to sTI-RADS and 1 point was assigned to the solid composition. For echogenicity, sTI-RADS included “hypoechoic” and assigned 1 point as very hypoechoic. For echogenic foci, sTI-RADS included peripheral calcifications and assigned 1 point for punctate echogenic foci. However, the ACR-TI-RADS assigned III was defined as 0 points, whereas the 2020 C-TI-RADS assigned III was defined as 1 point. In the sTI-RADS, the authors followed the 2017 ACR-TI-RADS scoring criteria and classified them into III, which was defined as 0 points (Fig. 4).

### The diagnostic performance of sTI-RADS, 2017 ACR-TI-RADS, and 2020 C-TI-RADS

Among the sTI-RADS, 2017 ACR-TI-RADS, and 2020 C-TI-RADS, the sTI-RADS had the highest specificity, accuracy, and positive predictive value (67.39%, 85.85%, and 86.68%, respectively), followed by the 2020 C-TI-RADS (64.78%, 85.58%, and 85.86%, respectively) and 2017 ACR-TI-RADS (46.09%, 82.91%, and 80.59%, respectively). The sTI-RADS had the highest Youden index (Table 4). In addition, the sTI-RADS had the maximum AUC (0.867), followed by the 2020 C-TI-RADS (0.865) and 2017 ACR-TI-RADS (0.861) (Fig. 3).

**Table 4:** Comparison of diagnostic efficiency in two guidelines and sTI-RADS

	Sen (%)	Spe (%)	Accuracy (%)	PPV (%)	NPV (%)	Youden index
ACR-TI-RADS	99.23	46.09	82.91	80.59	96.36	0.45
C-TI-RADS	94.80	64.78	85.58	85.86	84.66	0.60
sTI-RADS	94.03	67.39	85.85	86.68	83.33	0.61

*PPV;* Positive predictive value *NPV*; Negative predictive value *Sen*; Sensitivity *Spe*; Specificity

### Supplement to sTI-RADS

**Fig. 5:**
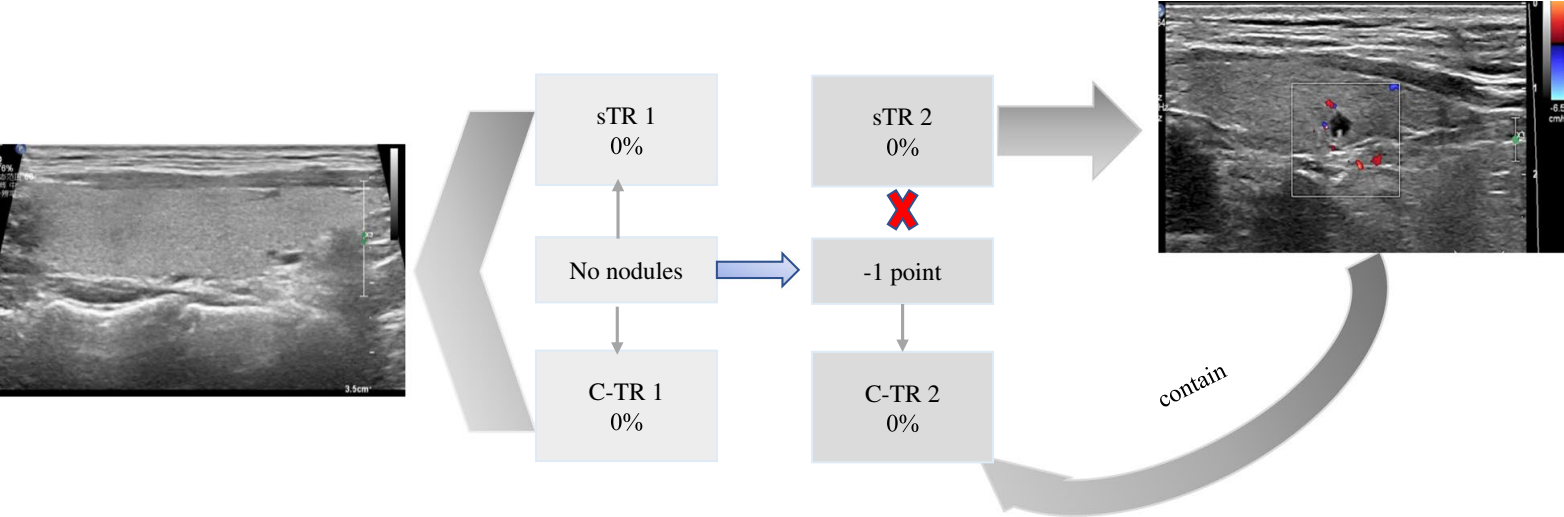
There was absence of nodules in the thyroid gland classified into as sTI-RADS 1 and 2020 C-TI-RADS 1. And the glial cysts are classified into as sTI-RADS 2, while -1 point was classified into as 2020 C-TI-RADS 2

According to the thyroid nodule sTI-RADS score, there was a lack of classification of sTI-RADS 1 and sTI-RADS 2. Therefore, the authors classified the absence of nodules in the thyroid gland as sTI-RADS 1 with a malignancy rate of 0% and glial cysts as sTI-RADS 2 with a malignancy rate of 0%. Compared with the 2020 C-TI-RADS, the assignment result of -1 point was no longer applicable (Fig. 5).

### Consistency of test results

FNAB or surgery confirmed which of 123 thyroid nodules were malignant (*n* = 90) or benign (*n* = 33). The ICC values of the 2017 ACR-TI-RADS, 2020-C-TI-RADS, and sTI-RADS were all greater than 0.80. Therefore, the intragroup diagnostic consistency of the five ultrasound doctors was good (Table 5). When comparing the diagnostic consistency between ultrasound doctors at the same level or with the same title, the kappa value of sTI-RADS was the highest, indicating that the intergroup diagnostic consistency was good only for sTI-RADS. The sTI-RADS had the highest accuracy, PPV, and Youden index; the Youden index showed an upward trend with an increase in the seniority of ultrasound doctors. A comparison of the diagnostic efficacy of the three scoring systems among the five ultrasound doctors is shown in Table 5.

**Table 5:** Comparison of diagnostic efficacy of different grading systems among five doctors

Sen (%)		Spe (%)	Accuracy (%)	PPV (%)	NPV (%)	Youden index	ICC	Kappa
C-TI-RADS							0.84 (0.78–0.88)	
A	84.44	87.88	85.37	95.00	67.44	0.72		
B1	77.78	93.94	82.11	97.22	60.78	0.72		0.69^K1^
B2	81.11	78.79	80.49	91.25	60.47	0.60		
C1	85.56	87.88	86.18	95.06	69.05	0.73		0.71^K2^
C2	84.44	90.91	86.18	96.20	68.18	0.75		
ACR-TI-RADS							0.86 (0.82–0.90)	
A	98.89	54.55	86.99	85.58	94.74	0.53		
B1	98.89	42.42	83.74	82.41	93.33	0.41		0.68^K1^
B2	98.89	60.61	88.62	87.25	95.24	0.60		
C1	97.78	72.73	91.06	90.72	92.31	0.71		0.67^K2^
C2	98.89	66.67	90.24	89.00	95.65	0.66		
sTI-RADS							0.88 (0.84–0.91)	
A	97.78	90.91	95**.**93	96.70	93.75	0.89		
B1	95.56	93.94	95.12	97.73	88.57	0.90		0.78^K1^
B2	95.56	84.85	92.68	94.51	87.50	0.80		
C1	92.22	84.85	90.24	94.32	80.00	0.77		0.73^K2^
C2	94.44	81.82	91.06	93.41	84.38	0.76		

A represents pro. Zhang; B1 represents attending doctor Cheng; B2 represents attending doctor Li; C1 represents residency Zheng; C2 represents residency Zhang. K1 represents the Kappa value of B1 and B2; K2 represents the Kappa value of C1 and C2;

## Discussion

In this study, a simplified version of the TI-RADS was proposed, namely, the sTI-RADS. It had the highest specificity, accuracy, positive predictive value, and Youden index compared with the 2017 ACR-TI-RADS and 2020 C-TI-RADS. The authors calculated the OR value of each ultrasonic sign in 749 nodules, which was based on the nature of the thyroid nodules [17–20]. According to the results, the authors reassigned the ultrasonic signs as 1 point with an OR > 1, whereas the others were reassigned as 0 points (OR < 1).

Compared with the 2017 ACR-TI-RADS, the sTI-RADS has the following advantages. First, the latter simplified the process of assignment. Those ultrasonic signs that were assigned 2 or 3 points under the 2017 ACR-TI-RADS were assigned 1 point under the sTI-RADS, and ultrasonic signs that were assigned 1 or 0 points under the 2020 C-TI-RADS were assigned 0 points with sTI-RADS. Another advantage is that sTI-RADS increased the number of subgroups in category 4 of ACR-TI-RADS, namely, sTI-RADS 4a, sTI-RADS 4b, and sTI-RADS 4c. In addition, the sTI-RADS enriched the detailed malignancy rate under various grades, including the following: sTI-RADS 3 (thyroid nodule score of 0 points) with a malignancy rate < 2%; sTI-RADS 4a (thyroid nodule score of 1 point) with a malignancy rate of 2–10%; sTI-RADS 4b (thyroid nodule score of 2 points) with a malignancy rate of 10–50%; sTI-RADS 4c (thyroid nodule score of 3 points) with a malignancy rate of 50–90%; and sTI-RADS 5 (thyroid nodule score of 4 and 5 points) with a malignancy rate > 90%, which was similar to the 2020 C-TI-RADS. Surprisingly, the sTI-RADS had the highest specificity (67.39%), indicating that the sTI-RADS could identify nonpatients more accurately than the 2017 ACR-TI-RADS and 2020 C-TI-RADS.

Compared with the 2020 C-TI-RADS, the sTI-RADS has some advantages. It enriches the ultrasonic signs of the assignment process; for echo, the C-TI-RADS only assigned the very hypoechoic as 1 point, whereas the sTI-RADS assigned both the hypoechoic and very hypoechoic as 1 point. Although some scholars have proposed that the malignancy risk of a markedly hypoechoic thyroid nodule is higher than that of a hypoechoic nodule [21, 22], a solid hypoechoic nodule with any suspicious US features (microcalcification, taller than wide shape, and spiculated/microlobulated margin) has a high malignancy risk [19]. In addition, the sTI-RADS has abandoned the -1 point of the comet tail artifacts and assigned it as 0 points; thus, the negative points were removed.

In this study, five ultrasound doctors with different qualifications evaluated the diagnostic efficacy of the three scoring systems using 123 thyroid nodules in a double-blind manner. The ICC values of the 2017 ACR-TI-RADS, 2020 C-TI-RADS, and sTI-RADS were greater than 0.80, indicating good diagnostic consistency among the different ultrasound doctors. To avoid selection bias, the authors tested the diagnostic consistency of ultrasound doctors at the same qualification level using Kappa. The Kappa values were the highest for attending doctors CLG and LR and residents ZS and ZYK, indicating that the intergroup diagnostic consistency was good only for sTI-RADS. However, in the evaluation of the 2017 ACR-TI-RADS and 2020 C-TI-RADS, the Kappa value was lower than 0.70, and diagnostic consistency among doctors was rated as medium. In terms of diagnostic accuracy, Table 5 shows that the diagnostic accuracy of different doctors through sTI -RADS was more than 90%, which demonstrates obvious advantages compared with the 2017 ACR-TI-RADS and 2020 C-TI-RADS. The sTI-RADS also had the largest Youden index. As the ultrasound doctors’ qualifications and working years increased, the Yoden index showed an upward trend; that is, the reliability of the diagnosis increased.

In this study, the proportion of malignant thyroid nodules was as high as 69.84% (609/872), which is much higher than previously reported in the literature. The main reasons for this are as follows. First, almost all of the patients with thyroid nodules were evaluated using conventional ultrasonography before puncture or surgery. For nodules with benign or a high possibility of benign nodules, further examination or treatment was not recommended. Second, for thyroid nodules suspected of malignancy, contrast-enhanced ultrasonography was performed to further evaluate the nature of the nodules. For nodules that were benign or highly likely to be benign after contrast-enhanced examination, it was suggested that the patients follow up for further observation. Third, all post-ultrasound guided biopsy thyroid nodules were excluded from this study. Cytological nodules classified as class I, III, IV, and V were excluded. Although a large proportion of these nodules were benign, they were excluded due to the lack of clear pathological diagnosis.

### Study limitations

Our study has some limitations. Thyroid nodule scoring ranged from 0 to 5 points, which corresponded to the grading system of sTI-RADS 3 to 5 only. We then assigned no nodules in the thyroid gland as sTI-RADS 1 and glial cysts as sTI-RADS 2, both of which had a malignancy rate of 0%. This designation method was not rigorous; however, the assignment method was convenient, and the nodule score was overlaid. This was a single-center, large-sample study, therefore, we hope to obtain repeated studies from more research centers.

## Conclusion

In summary, a new version of TI-RADS was proposed, namely, sTI-RADS. Through the practice of five ultrasound doctors with different qualifications, it was confirmed that the sTI-RADS has higher specificity, accuracy, PPV, and Youden index than the 2017 ACR-TI-RADS and 2020 C-TI-RADS. Given these advantages, this study provides strong evidence that the sTI-RADS would provide great benefits to the clinical settings in which it is utilized.

## Data Availability

The datasets generated and/or analyzed during the current study are available from the corresponding author upon reasonable request.

## Abbreviations

TC: Thyroid cancer
PTMC: Papillary thyroid microcarcinoma
FNAB: Fine-needle aspiration biopsy
ACR-TI-RADS: American College of Radiology-Thyroid Imaging Reporting and Date System
C-TI-RADS: Chinese -Thyroid Imaging Reporting and Date System
sTI-RADS: Simplified Thyroid Imaging Reporting and Date System
ROC: Receiver operating characteristic curve
